# NHERF1 and tumor microenvironment: a new scene in invasive breast carcinoma

**DOI:** 10.1186/s13046-018-0766-7

**Published:** 2018-05-02

**Authors:** Concetta Saponaro, Alessandro Vagheggini, Emanuela Scarpi, Matteo Centonze, Ivana Catacchio, Ondina Popescu, Maria Irene Pastena, Francesco Giotta, Nicola Silvestris, Anita Mangia

**Affiliations:** 1 Functional Biomorphology Laboratory, IRCCS-Istituto Tumori “Giovanni Paolo II”, 70124 Bari, Italy; 2 Unit of Biostatistics and Clinical Trials, Istituto Scientifico Romagnolo per lo Studio e la Cura dei Tumori (IRST)-IRCCS, 47014 Meldola, FC Italy; 3 Pathology Department, IRCCS-Istituto Tumori “Giovanni PaoloII”, 70124 Bari, Italy; 4 Medical Oncology Unit, IRCCS-Istituto Tumori “Giovanni Paolo II”, 70124 Bari, Italy; 5 Scientific Direction, IRCCS-Istituto Tumori “Giovanni Paolo II”, 70124 Bari, Italy

**Keywords:** Tumor microenvironment, Tissue microarray, Immunohistochemical, NHERF1, Breast cancer

## Abstract

**Background:**

Tumor microenvironment (TME) includes many factors such as tumor associated inflammatory cells, vessels, and lymphocytes, as well as different signaling molecules and extracellular matrix components. These aspects can be de-regulated and consequently lead to a worsening of cancer progression. In recent years an association between the scaffolding protein Na^+^/H^+^ exchanger regulatory factor 1 (NHERF1) and tumor microenvironment changes in breast cancer (BC) has been reported.

**Methods:**

Subcellular NHERF1 localization, vascular endothelial growth factor (VEGF), its receptor VEGFR1, hypoxia inducible factor 1 alpha (HIF-1α), TWIST1 expression and microvessel density (MVD) in 183 invasive BCs were evaluated, using immunohistochemistry on tissue microarrays (TMA). Immunofluorescence was employed to explore protein interactions.

**Results:**

Cytoplasmic NHERF1(cNHERF1) expression was directly related to cytoplasmic VEGF and VEGFR1 expression (*p* = 0.001 and *p* = 0.027 respectively), and inversely to nuclear HIF-1α (*p* = 0.021) and TWIST1 (*p* = 0.001). Further, immunofluorescence revealed an involvement of tumor cells with NHERF1 positive staining in neo-vascular formation, suggesting a “mosaic” structure development of these neo-vessels. Survival analyses showed that loss of nuclear TWIST1 (nTWIST1) expression was related to a decrease of disease free survival (DFS) (*p* < 0.001), while nTWIST1-/mNHERF1+ presented an increased DFS with respect to nTWIST1+/mNHERF1- phenotype (*p* < 0.001). Subsequently, the analyses of nTWIST1+/cNHERF1+ phenotype selected a subgroup of patients with a worse DFS compared to nTWIST1-/cNHERF1- patients (*p* = 0.004).

**Conclusion:**

Resulting data suggested a dynamic relation between NHERF1 and TME markers, and confirmed both the oncosuppressor role of membranous NHERF1 expression and the oncogene activity of cytoplasmic NHERF1.

**Electronic supplementary material:**

The online version of this article (10.1186/s13046-018-0766-7) contains supplementary material, which is available to authorized users.

## Background

Tumor microenvironment (TME) is a complex system in which different cell types, playing an important role in carcinogenesis and tumor-host relationship, co-exist. A bidirectional communication between cells and microenvironment has been highlighted in normal tissue homeostasis and also in the tissues involved in different diseases [1]. In this context the tumor generates and proliferates, surrounded by blood vessels, fibroblasts, inflammatory and immune cells, lymphocytes, different signaling molecules and extracellular matrix. Thus, the tumor can directly affect the microenvironment by the release of extracellular signals and immune escape mechanisms [2]. The interactions are often intricate and their characterization could permit to identify prognostic and predictive biomarkers useful for the development of new pharmacological targets and to provide novel therapeutical opportunities for cancer patients. This is also true for breast cancer (BC), one of the most diffused cancer in women, with a high incidence rate in all countries [3]. Recently, the heterogeneity of cellular and non-cellular components of TME has been underlined, including growth factors, cytokines, RNA, DNA, metabolites and matricellular proteins. They are involved in cancer onset and sustenance by supplying metabolites, energy, drug resistance environment, metastatic, growth and angiogenetic signals as well as evading immune surveillance [4, 5]. Alterations and local changes in the concentration of angiogenic factors such as vascular endothelial growth factor (VEGF), its receptor (VEGFR1) and transcriptional targets of hypoxia inducible factor 1 alpha (HIF-1α), may trigger angiogenesis, by increasing microvessel density (MVD) in hypoxic breast TME [6, 7]. Among the factors distinguishing the TME, the epithelial-mesenchymal transition (EMT) plays an important role as well, and to date the involvement of some EMT markers has been extensively investigated [8]. TWIST1 is a basic helix-loop-helix transcription factor that contributes to carcinogenesis by triggering EMT, thereby influencing tumor invasion behavior [9]. TWIST1 over-expression was confirmed as having an essential role in tumor initiation, stemness, angiogenesis, invasion, metastasis, and chemo-resistance in a variety of carcinomas [10–13].

During recent years, our focus has been on the adaptor protein Na^+^/H^+^ exchanger regulating factor 1 (NHERF1), a scaffold protein, with two PDZ and one EB (Ezrin-binding) domains, able to link different molecules and to drive several signal transduction pathways [14]. Its implication in different tumors such as predictive/prognostic biomarkers has been extensively investigated [15–17], but little is known about NHERF1-TME relation. Our previous studies underlined its possible involvement in the orchestrate TME signaling pathway. In fact, its overexpression is implicated in the increase of invasive phenotype in breast cancer cells, in synergy with exposure to the hypoxic tumor microenvironment and with HIF-1α expression [18]. Furthermore, at membranous and cytoplasmic level, its overexpression and co-localization with VEGFR1, a marker correlated with high metastasis risk and relapse has been evaluated [19]. A similar behavior has been also observed in advanced colorectal cancer (CRC), where overexpression of nuclear NHERF1 in association with hypoxic microenvironment and tumor invasive phenotype has been reported [20]. In a recent study, NHERF1 expression was positively correlated with VEGFR2 expression in CRC, suggesting NHERF1 involvement in disease progression by VEGFR2 signaling pathway [21]. The present study aimed to evaluate the possible involvement of NHERF1 and its association with the major TME markers as VEGF, VEGFR1, HIF-1α, MVD and TWIST1, in 183 invasive BC tumors. Moreover, we investigated possible specific immunophenotypes useful in identifying a subgroup of BC patients with a different clinical outcome, related to these TME markers. A better characterization of TME could allow the identification of new combinations of prognostic biomarkers valuable for clinical management and the development of novel therapeutic strategies.

## Methods

### Patients and tissues

The tissue microarray (TMA) utilized in this retrospective study included 549 samples of invasive BCs from 183 patients. The patients were subjected to primary surgery with nodal dissection at the Institute IRCCS Istituto Tumori “Giovanni. Paolo II” of Bari, Italy, in the years 2002–2003. For each tumor, 3 punches of cancerous tissues were available. The median age of patients was 49 years (range 24–83). For the subgroup of patients with follow-up (64,5%), the median follow-up time was 82 months (range 1–162 months). TNM classification, tumor size, grade, perineoplastic invasion, estrogen receptor (ER), progesterone receptor (PgR), proliferative activity (MIB1) and HER2 status were provided by the Pathology Department of our Institute. The clinicopathological characteristics of the patients are listed in Table 1. Tumors with ER or PgR expression were classified as positive when nuclear staining was found in > 10%. MIB1 nuclear staining was used to assess the proliferative activity, with a cut off value of 20% positive cells to indicate the tumors with MIB1 >  20% as highly proliferating. This cut off is the median value of scores relative to all breast tumors samples analysed during these years within our Institute. HER2 protein expression was studied using a monoclonal antibody (MoAb clone CB11; Novocastra Laboratories, Ltd., Newcastle, UK) and scored in accordance with the HercepΤest scoring system (Food and Drug Administration): 0 indicated no membranous immunoreactivity or < 10% of cells reactive; 1+, an incomplete membranous reactivity in > 10% of cells; 2+, ≥10% of cells with weak to moderate complete membranous reactivity; and 3+, strong and complete membranous reactivity in > 10% of cells. Cytoplasmic immunoreactivity was ignored. Cases scoring 0 and 1+ were classified as negative. HER2 was considered to be positive if immunostaining was 3+ or if a score 2+ showed gene amplification by fluorescence in situ hybridization (FISH). In FISH analyses, each copy of the HER2 gene and its centromere 17 (CEP17) reference was counted. The interpretation was in accordance with the criteria of 2007 ASCO/CAP guidelines for HER2 testing in BC, therefore positive if the HER2/CEP17 ratio was higher than 2,2 [22].

**Table 1:** Tumor characteristics of 183 invasive breast cancer patients

Characteristics	*n*	(%)
*Age*: median value 49 (range 24–83)		
≤ 49 years	89	(48.6)
> 49 years	94	(51.4)
*Histological type*		
IDC	116	(90.7)
ILC	10	(5.5)
Other	7	(3.8)
*Histological grade*		
G1	29	(15.8)
G2	84	(45.9)
G3	70	(38.3)
*Tumor size (cm)*		
≤ 2 cm	81	(44.8)
> 2 cm	100	(55.2)
Unknown	2	
*Lymph node status*		
Negative	71	(40.8)
Positive	103	(59.2)
Unknown	9	
*Estrogen receptor*		
ER-negative (≤10%)	53	(29.0)
ER-positive (> 10%)	130	(71.0)
Unknown	1	
*Progesterone receptor*		
PgR-negative (≤10%)	76	(41.5)
PgR-positive (> 10%)	107	(58.5)
*MIB1*		
Negative (≤20%)	77	(42.1)
Positive (> 20%)	106	(57.9)
*HER2/neu*		
Negative (0,1+)	78	(50.6)
Positive (3+)	76	(49.4)
Unknown	29	
*Triple negative tumors*	19	(10.4)
*Treatment*		
CT	27	(25.5)
HT	16	(15.1)
CT + HT	63	(39.6)
Unknown	77	

TMAs were constructed manually from formalin-fixed and paraffin-embedded tissues, according to standard procedures as previously described [15]. In brief, tumor target areas were selected from one donor block per patient, three 0.5 mm cores were punched out and transferred to the recipient TMA blocks. Each sample was arrayed in triplicate to minimize tissue loss and to overcome tumor heterogeneity, thus the three cores were representative of the whole specimen.

### Immunohistochemistry (IHC) and evaluation

Four μm-thick slices were cut from the TMA blocks and transferred to slides. The TMA slides were processed and stained by the indirect immunoperoxidase method [23]. In brief, TMA slides were deparaffinised and partially rehydrated through absolute ethanol and 95% ethanol series. Antigen retrieval was performed by the 0.01 M citrate buffer (pH 6.0) at 98 °C in a water bath from a minimum of 20 to a maximum of 45 min. The slides were then allowed to cool for 30 min and the endogenous peroxidase activity was blocked for 10 min with 3% H_2_O_2_. The primary antibodies, diluted in PBS/BSA 1%, were incubated on the slides at 4 °C overnight in a moist chamber. For anti-VEGFR1, 1 h incubation at room temperature was required. A polymer-based IHC detection system was used as the amplification system (EnVision + System-HRP Labelled Polymer Anti-Rabbit or Anti-Mouse secondary antibody, Dako, Carpinteria, CA, USA) according to the manufacture’s instruction. The bound antibody was visualized by incubating the sections in 3-amino-9-ethylcarbazole (AEC + Substrate Chromogen, Dako, Carpinteria, CA, USA) for 15 min, except for anti-CD34, which requires the use of 3,3′-diaminobenzidine (Liquid DAB + Substrate Chromogen System, Dako, Carpinteria, CA, USA) for 8–10 min. Cell nuclei were counterstained with Mayer’s Haematoxylin (Bio-Optica, MI, Italy) and the slides were mounted with aqueous mounting medium (Faramount Aqueous Mounting Medium, Dako, Carpinteria, CA, USA). The different analysed biomarkers, dilution, source/clone, the staining localization of antibody and the cut off [median value or immunohistochemical score (HIS)] used to classify positive versus negative cases are shown in Additional file 1: Table S1.

All immunostained samples were scored by double-blinded independent observers who had no patient outcome and clinicopathological data information, and the mean of the three readings for each patient was calculated. If one core was uninformative, lost or contained no tumor tissue, the overall score applied was that of the remaining cores. The results from two observers were identical in most cases, and discrepancies were resolved by re-examination and consensus.

NHERF1 immunostaining was predominantly cytoplasmic (cNHERF1), however in some cases an intense nuclear (nNHERF1) and membranous (mNHERF1) staining were also demonstrated. These were scored separately and their significance was evaluated. VEGF and VEGFR1 were mainly observed in the cytoplasm of breast cancer tissues (cVEGF and cVEGFR1), and the staining was evaluated as HIS or percentage of immunoreactive cells, respectively. Nuclear localization of HIF-1α (nHIF-1α) and TWIST1 (nTWIST1) was mainly observed. Cytoplasmic staining of HIF-1α and TWIST1 was occasionally reported even if it was not evaluated for the aims of the study. The median value of immunoreactive cells was used as cut off for cNHERF1 (≥40%), nNHERF1 (> 0%), mNHERF1 (> 0%), nTWIST1 (≥4%), cVEGFR1 (> 0%), nHIF-1α (> 0%) and MVD (≥15 microvessels/mm^2^). For cVEGF, the HIS was calculated by combining the quantity score (percentage of positive stained cells) with the staining intensity score [6]. The quantity score ranged from 0 to 4: 0 = no immunoreactivity; 1 ≤ 25% cells stained; 2 = 26–50% cells stained; 3 = 51–75% cells stained; and 4 = ≥76% cells stained. The staining intensity was scored as: 0 (negative), 1 (weak), 2 (moderate) and 3 (strong). Raw data were converted to IHS by adding the quantity score (0–4) to the staining intensity score (0–3). Theoretically, the scores can range from 0 to 7. An IHS of 6–7 was considered a strong immunoreactivity; 3–5, moderate; 1–2, weak; and 0, negative. For our analyses, tumors presenting a moderate or strong score were cVEGF positive (IHS:3–7). Finally, microvessel counting was performed by identifying the areas which represented the highest vascular density, the so called “hot spots”. The MVD measurements were made in the fields with a higher density of CD-34 positive cells and cell clusters at 200× magnification, as previously described [6]. Positive and negative controls were included in each staining run as indicated in the data sheet of each antibody. The accuracy, reliability, scoring strategy and reproducibility assessments of these antibodies have been validated in previous studies already published [6, 24–26].

### Immunofluorescence

Immunofluorescent analysis was performed as described previously [18]. Briefly, formalin-fixed and paraffin embedded tissue serial sections of 3 μm in thickness were deparaffinized with xylene, and rehydrated in an ethanol series. Antigen retrieval was carried out immersing slide in a 0.01 M saline citrated buffer (pH 6.0) at 95 °C for 40 min, then tissues were permeabilized with 0.1% Triton X100-Phosphate Buffered Saline for 15 min, blocked 30 min with 1% Bovine Serum Albumin-Phosphate Buffered Saline and incubated overnight at 4 °C in a humidified chamber with a mouse monoclonal anti-CD34 (clone QBEND-10, dilution 1:20; Novocastra Lab. Ltd., UK) together with a rabbit polyclonal anti-NHERF1 (PA1–090, 1 μg/100 μL dilution; Affinity Bio-Reagents). The slides were then incubated at room temperature for 1 h with the Alexa Fluor 488 and Alexa Fluor 568 immunoglobulin G secondary conjugated antibodies (1:2000 dilution; Molecular Probes Inc., Eugene, OR, USA) and mounted with DAPI (ProLong® Gold antifade reagent; Molecular Probes Inc.). Positive control slides that were run simultaneously were used for assessing the quality of immunoreactivity. For negative controls, slide sections that were immunopositive were treated with 1% Bovine Serum Albumin instead of the primary antibody, and no reactivity was observed in any of these controls. Images were obtained on a BX40 microscope (Olympus, Tokyo, Japan) with a SenSys 1401E-Photometrics charge-coupled device camera. To verify protein colocalization, each acquired stack was merged by transforming the three channels corresponding to red (tetramethylrhodamine B isothiocyanate), green (fluorescein isothiocyanate) and blue (4′,6-diamidino-2-phenylindole) into a single three-color stack by using the “RGB merge” command of ImageJ software (National Institutes of Health Bethesda, MD).

### Follow up and statistical analysis

Associations of tumor markers expression with various clinicopathological features and the among markers themselves were determined by the Chi-squared test for the independence of categorical variables. The presence of non-linear relationship between two continuous variables was assessed by the Kendall rank test.

The results from the immunohistochemical analyses of our biomarkers were assessed in relation to disease-free survival (DFS) and overall survival (OS). DFS (in months) was defined as the time from diagnosis to the date of loco-regional or distant recurrence, second invasive breast carcinoma, second primary cancer and/or death without evidence of breast cancer or to the date of last contact. OS (in months) was defined as the time from diagnosis to the date of last contact or of death from any cause. Univariate analyses of DFS and OS were carried out considering both tumor markers expression and clinicopathological variables: five-year DFS and OS and their 95% confidence interval were based on Kaplan-Meier method, hazard-ratio (HR) were computed using the Cox proportional hazard regression model for DFS only (for OS it could not be computed due to the low number of deaths), and *p-values* of the log-rank test were used to infer on the equality of probability of an event (relapse for DFS or death for OS) in the two groups. Multivariate analyses on DFS (OS could not be analyzed due to the low number of deaths) were performed for tumor markers expressed as categorical and continuous variables using the Cox proportional hazard regression model; HRs were computed along with their *p-values*.

Data analysis was carried out using the statistical packages survival and survminer of the statistical language R, version 3.4 [27].

## Results

### Clinicopathological characteristics

The tumor characteristics of the 183 patients included in the study are listed in Table 1. The 48.6% of patients were younger than 49 years. The majority had an invasive ductal carcinoma (IDC) (90.7%), a moderate histological grade (G2) (45.9%), and tumors larger than 2 cm (55.2%) with axillary lymph node involvement (59.2%). Furthermore, most patients had ER (71%) and PgR (58.5%) positive status, high MIB1 (57.9%) and HER2/neu negative (50.6%); 10.4% were triple negative breast cancers. The treatment of only 106 patients was known. Of these, 25.5% received adjuvant chemotherapy, 15.1% of ER-positive patients received hormonotherapy, and 39.6% received adjuvant tamoxifen for 5 years after the end of chemotherapy.

### Protein expression profiling

**Fig. 1:**
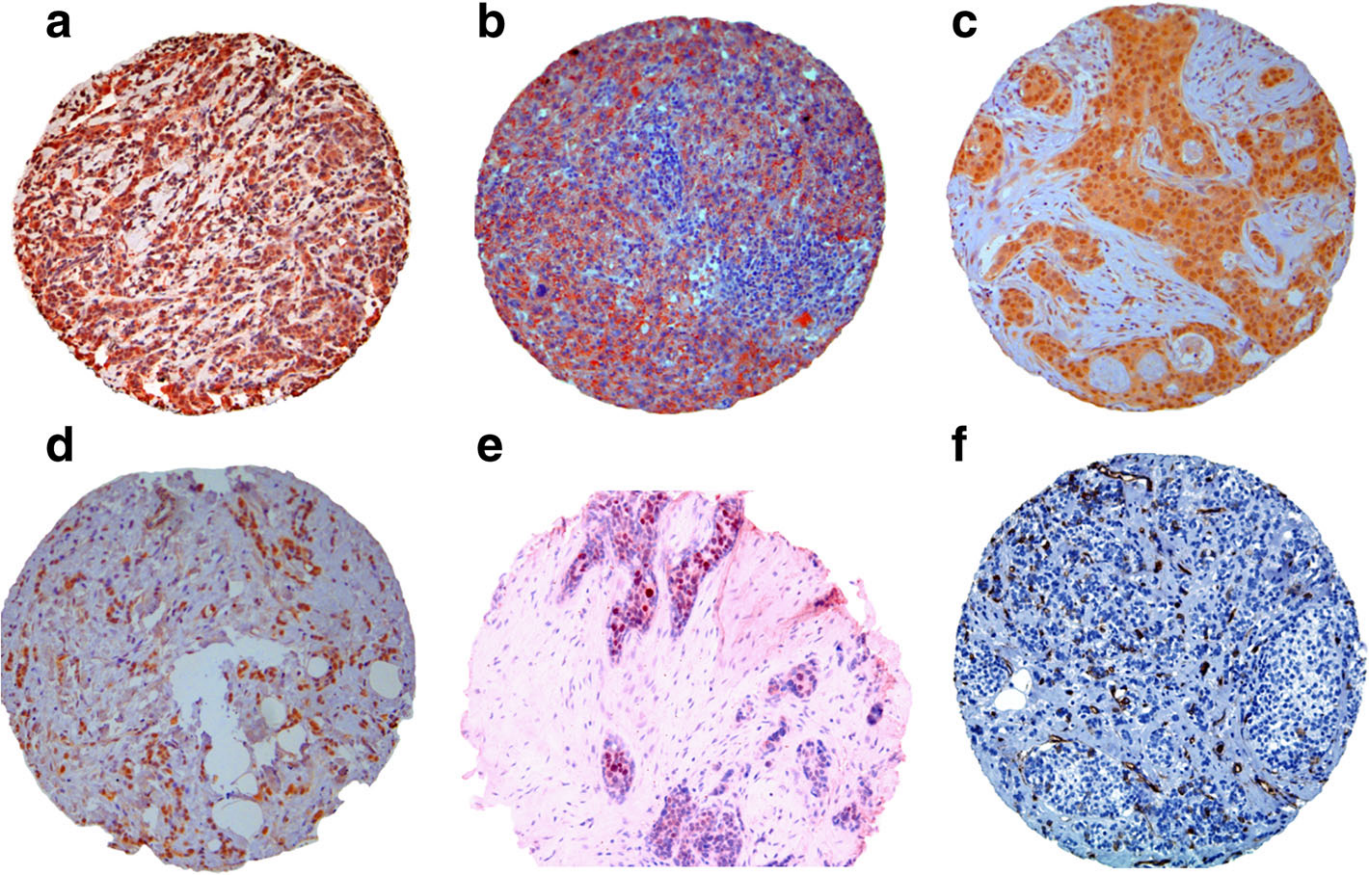
Immunohistochemical expression TME biomarkers on tissue microarrays. Representative images of immunohistochemical staining of TMA tumor cores for invasive breast tumor cells, 100× magnification, with **a** cytoplasmic NHERF1 and nuclear NHERF1 expression; **b** high cytoplasmic VEGF expression; **c** high cytoplasmic VEGFR1 expression; **d** nuclear HIF-1α expression; **e** nuclear TWIST1 expression and **f** high microvessel density (MVD)

The immunohistochemical expression of NHERF1, TWIST1, VEGFR1, VEGF, HIF-1α and MVD, was evaluated on TMA sections. The frequency of expression of these biomarkers, in the whole cohort of tumor samples is reported in Additional file 2: Table S2. NHERF1 expression was detected in the apical membrane, cytoplasm, and nucleus of tumor samples. These different localizations were scored separately and their significance was evaluated. All breast cancers showed NHERF1 protein localized in the cytoplasm of tumor cells, of these 49% (76/154) overexpressed cNHERF1, according to the median value ≥40%. Membranous NHERF1 and nNHERF1expression were detected in 13% (20/151) and 20% (30/149) of cases, respectively (median value > 0 for both compartments). Cytoplasmic VEGF was expressed in 96% (144/150) of tumor cells, of these 54% (81/151) overexpressed it, according to the IHS median value ≥3. Cytoplasmic VEGFR1 and nuclear HIF1α were expressed in about 50% (80/161) and 37% (56/153) of tumor cells and, according to the median value > 0 for both markers. Nuclear TWIST1 was expressed in 60% (84/140) of tumor cells, of these 51% (72/140) overexpressed it, according to the median value ≥4. Tumors showed microvessel presence and 54% (85/157) of them were characterized by a higher MVD, according to the median value ≥15 microvessels/mm^2^. Figure 1 shows an example of immunohistochemical staining pattern of NHERF1 and VEGF, VEGFR1, HIF-1α, TWIST1 and MVD expression in TMA tumor cores. NHERF1 was present in cytoplasmic compartment and less frequently at nuclear level, according with the median values of expression. VEGF, VEGFR1, HIF-1α, TWIST1 and MVD expression was heterogeneous for intensity and expression patterns in the different tumor samples examined.

### Relationship between tumor markers and clinicopathological features

A summary of significant association between tumor marker expressions and clinicopathological features are reported in Table 2. Among the BCs with cNHERF1 overexpression, 67.1% exhibited a significant association with tumor size > 2 cm (*p* = 0.023) and 68.4% with MIB1 positive expression (*p* = 0.027). Further, negative cNHERF1 expression was related to PgR-positive expression (*p* = 0.023) and HER2/neu negative (*p* = 0.002) in 67.1% and 65.2% of cases respectively. The lack of nuclear NHERF1 was noticeably associated to tumor size > 2 cm (*p* = 0.014), while its presence was linked to MIB1 negative expression (*p* < 0.001). Analyzing NHERF1 reactivity at membrane level, its absence was observed in IDCs (p = 0.002) and positive lymph node status (*p* = 0.013); conversely mNHERF1 expression was significantly associated with tumor size ≤2 cm (*p* = 0.039) and MIB1 negative (*p* = 0.019). A statistically significant relation was observed between mNHERF1 and PgR-positive expression (*p* = 0.034).

**Table 2:** Relationship between tumor markers and clinicopathological features

	*cNHERF1*					*nNHERF1*					*mNHERF1*					*cVEGF*				
	Negative		Positive		*p-value*	Negative		Positive		*p-value*	Negative		Positive		*p-value*	Negative		Positive		*p-value*
Characteristics	n	(%)	n	(%)		n	(%)	n	(%)		n	(%)	n	(%)		n	(%)	n	(%)	
*Age*																				
≤ 49 years	38	(52.1)	40	(52.6)	*0.944*	63	(52.9)	15	(50.0)	*0.773*	70	(53.4)	10	(50.0)	*0.774*	27	(39.1)	50	(61.7)	***0.006***
> 49 years	35	(47.9)	36	(47.4)		56	(47.1)	15	(50.0)		61	(46.6)	10	(50.0)		42	(60.9)	31	(38.3)	
*Histological type*																				
IDC	66	(90.4)	72	(94.7)	*0.511*	111	(93.3)	27	(90.0)	*0.820*	125	(95.4)	15	(75.0)	***0.002***	59	(85.5)	78	(96.3)	*0.051*
ILC	3	(4.1)	1	(1.3)		3	(2.5)	1	(3.3)		3	(2.3)	1	(5.0)		4	(5.8)	2	(2.5)	
Other	4	(5.5)	3	(3.9)		5	(4.2)	2	(6.7)		3	(2.3)	4	(20.0)		6	(8.7)	1	(1.2)	
*Tumor size (cm)*																				
≤ 2 cm	37	(51.4)	25	(32.9)	***0.023***	44	(37.0)	18	(62.1)	***0.014***	50	(38.2)	12	(63.2)	***0.039***	33	(47.8)	28	(35.0)	*0.112*
> 2 cm	35	(48.6)	51	(67.1)		75	(63.0)	11	(37.9)		81	(61.8)	7	(36.8)		36	(52.2)	52	(65.0)	
*Lymph node status*																				
Negative	24	(34.3)	30	(41.7)	*0.365*	42	(36.8)	12	(42.9)	*0.557*	42	(33.6)	12	(63.2)	***0.013***	33	(51.6)	24	(30.4)	***0.010***
Positive	46	(65.7)	42	(58.3)		72	(63.2)	16	(57.1)		83	(66.4)	7	(36.8)		33	(48.4)	55	(69.6)	
*Histological grade*																				
G1	15	(20.5)	11	(14.5)	*0.086*	17	(14.3)	9	(30.0)	*0.077*	20	(15.3)	6	(30.0)	*0.138*	17	(24.6)	7	(8.6)	***0.020***
G2	38	(52.1)	31	(40.8)		55	(46.2)	14	(46.7)		60	(45.8)	10	(50.0)		30	(43.5)	37	(45.7)	
G3	20	(27.4)	34	(44.7)		47	(39.5)	7	(23.3)		51	(38.9)	4	(20.0)		22	(31.9)	37	(45.7)	
*Receptor status*																				
ER-negative (≤ 10%)	20	(27.4)	23	(30.3)	*0.700*	38	(31.9)	5	(16.7)	*0.099*	39	(29.8)	4	(20.0)	*0.367*	13	(18.8)	32	(39.5)	***0.006***
ER-positive (> 10%)	53	(72.6)	53	(69.7)		81	(68.1)	25	(83.3)		92	(70.2)	16	(80.0)		56	(81.2)	49	(60.5)	
PgR-negative (≤ 10%)	24	(32.9)	39	(51.3)	***0.023***	55	(46.2)	8	(26.7)	*0.053*	59	(45.0)	4	(20.0)	***0.034***	27	(39.1)	38	(46.9)	*0.338*
PgR-positive (> 10%)	49	(67.1)	37	(48.7)		64	(53.8)	22	(73.3)		72	(55.0)	16	(80.0)		42	(60.9)	43	(53.1)	
*MIB1*																				
Negative (≤ 20%)	36	(49.3)	24	(31.6)	***0.027***	39	(32.8)	21	(70.0)	***< 0.001***	49	(37.4)	13	(65.0)	***0.019***	40	(58.0)	23	(28.4)	***< 0.001***
Positive (> 20%)	37	(50.7)	52	(68.4)		80	(67.2)	9	(30.0)		82	(62.6)	7	(35.0)		29	(42.0)	58	(71.6)	
*HER2/neu*																				
Negative (0, 1+)	43	(65.2)	27	(38.0)	***0.002***	51	(81.4)	19	(65.5)	*0.080*	60	(49.2)	10	(58.8)	*0.456*	38	(63.3)	30	(39.5)	***0.006***
Positive (3+)	23	(34.8)	44	(62.0)		57	(18.6)	10	(34.5)		62	(50.8)	7	(41.2)		22	(36.7)	46	(60.5)	
	*cVEGFR1*					*MVD*					*nH1F-1α*					*nTWIST1*				
	Negative		Positive		*p-value*	Negative		Positive		*p-value*	Negative		Positive		*p-value*	Negative		Positive		*p-value*
Characteristics	n	(%)	n	(%)		n	(%)	n	(%)		n	(%)	n	(%)		n	(%)	n	(%)	
*Patient age*																				
≤ 49 years	43	(53.1)	41	(51.2)	*0.816*	35	(48.6)	43	(50.6)	*0.805*	50	(51.5)	30	(53.6)	*0.809*	36	(52.9)	35	(48.6)	*0.609*
> 49 years	38	(46.9)	39	(48.8)		37	(51.4)	42	(49.4)		47	(48.5)	26	(46.4)		32	(47.1)	37	(51.4)	
*Histological type*																				
IDC	71	(87.7)	77	(96.2)	***0.041***	68	(94.4)	76	(89.4)	*0.514*	91	(93.8)	50	(89.3)	*0.502*	63	(92.6)	66	(91.7)	*0.663*
ILC	6	(7.4)	0	(0.0)		2	(2.8)	4	(4.7)		2	(2.1)	3	(5.4)		3	(4.4)	2	(2.8)	
Other	4	(4.9)	3	(3.8)		2	(2.8)	5	(5.9)		4	(4.1)	3	(5.4)		2	(2.9)	4	(5.6)	
*Tumor size (cm)*																				
≤ 2 cm	39	(48.8)	29	(36.2)	*0.110*	36	(50.0)	31	(36.9)	*0.100*	36	(37.1)	29	(52.7)	*0.062*	26	(38.8)	32	(44.4)	*0.501*
> 2 cm	41	(51.2)	51	(63.8)		36	(50.0)	53	(63.1)		61	(62.9)	26	(47.3)		41	(61.2)	40	(55.6)	
*Lymph node status*																				
Negative	33	(44.0)	27	(35.1)	*0.260*	26	(38.2)	32	(39.5)	*0.874*	31	(34.4)	26	(47.3)	*0.125*	25	(39.1)	27	(39.7)	*0.940*
Positive	42	(56.0)	50	(64.9)		42	(61.8)	49	(60.5)		59	(65.6)	29	(52.7)		39	(60.9)	41	(60.3)	
*Histological grade*																				
G1	14	(17.3)	11	(13.8)	*0.246*	12	(16.7)	15	(17.6)	*0.898*	13	(13.4)	12	(21.4)	***0.001***	10	(14.7)	14	(19.4)	*0.251*
G2	40	(49.4)	32	(40.0)		32	(44.4)	40	(47.1)		34	(35.1)	32	(57.1)		26	(38.2)	34	(47.2)	
G3	27	(33.3)	37	(46.2)		28	(38.9)	30	(35.3)		50	(51.5)	12	(21.4)		32	(47.1)	24	(33.3)	
*Receptor status*																				
ER-negative (≤ 10%)	21	(25.9)	29	(36.2)	*0.157*	19	(26.4)	26	(30.6)	*0.562*	32	(33.0)	16	(28.6)	*0.570*	24	(35.3)	15	(20.8)	*0.056*
ER-positive (> 10%)	60	(74.1)	51	(63.8)		53	(73.6)	59	(69.4)		65	(67.0)	40	(71.4)		44	(64.7)	57	(79.2)	
PgR-negative (≤ 10%)	26	(32.1)	42	(52.3)	***0.009***	32	(44.4)	33	(38.8)	*0.476*	48	(49.5)	18	(32.1)	***0.037***	34	(50.0)	30	(41.7)	*0.323*
PgR-positive (> 10%)	55	(67.9)	38	(47.5)		40	(55.6)	52	(61.2)		49	(50.5)	38	(67.9)		34	(50.0)	42	(58.3)	
*MIB1*																				
Negative (≤ 20%)	37	(45.7)	29	(36.2)	*0.224*	32	(44.0)	34	(40.0)	*0.574*	31	(32.0)	29	(51.8)	***0.016***	22	(32.4)	38	(52.8)	***0.015***
Positive (> 20%)	44	(54.3)	51	(63.8)		40	(55.6)	51	(60.0)		66	(68.0)	27	(48.2)		46	(67.6)	34	(47.2)	
*HER2/neu*																				
Negative (0, 1+)	41	(58.6)	34	(44.7)	*0.095*	34	(54.0)	36	(48.0)	*0.485*	40	(44.9)	32	(61.5)	*0.057*	29	(46.8)	35	(50.7)	*0.652*
Positive (3+)	29	(41.4)	7	(55.3)		29	(46.0)	39	(52.0)		49	(55.1)	20	(38.5)		33	(53.2)	34	(49.3)	

*p-value* of Chi-squared test for the independence of categorical variables. Bold values indicate significance

High cVEGF expression was present in 61.7% of younger patients (*p* = 0.006), and it showed a significant association with positive lymph node status (*p* = 0.010), higher tumor histological grade (*p* = 0.020), MIB1 positive expression (*p* < 0.001). CytoplasmicVEGF negative detection was related to HER2/neu negative expression (*p* = 0.006). Furthermore, a statistically significant relation was observed between cVEGF negative and ER-positive expression (*p* = 0.006). Expression of cVEGFR1 was statistically prevalent in invasive tumor phenotype (*p* = 0.041) and PgR-positive expression (*p* = 0.009). G2 tumors presented high nHIF-1α expression (*p* = 0.001), and this over-expression was directly associated with PgR-positive expression (*p* = 0.037) while nHIF-1α negative expression was associated to MIB1 positive expression (*p* = 0.016). Low nTWIST1 expression was statistically prevalent in high MIB1 tumors (*p* = 0.015). There were no statistically significant associations between MVD and the clinicopathological features.

### Association between protein expressions analyzed

**Fig. 2:**
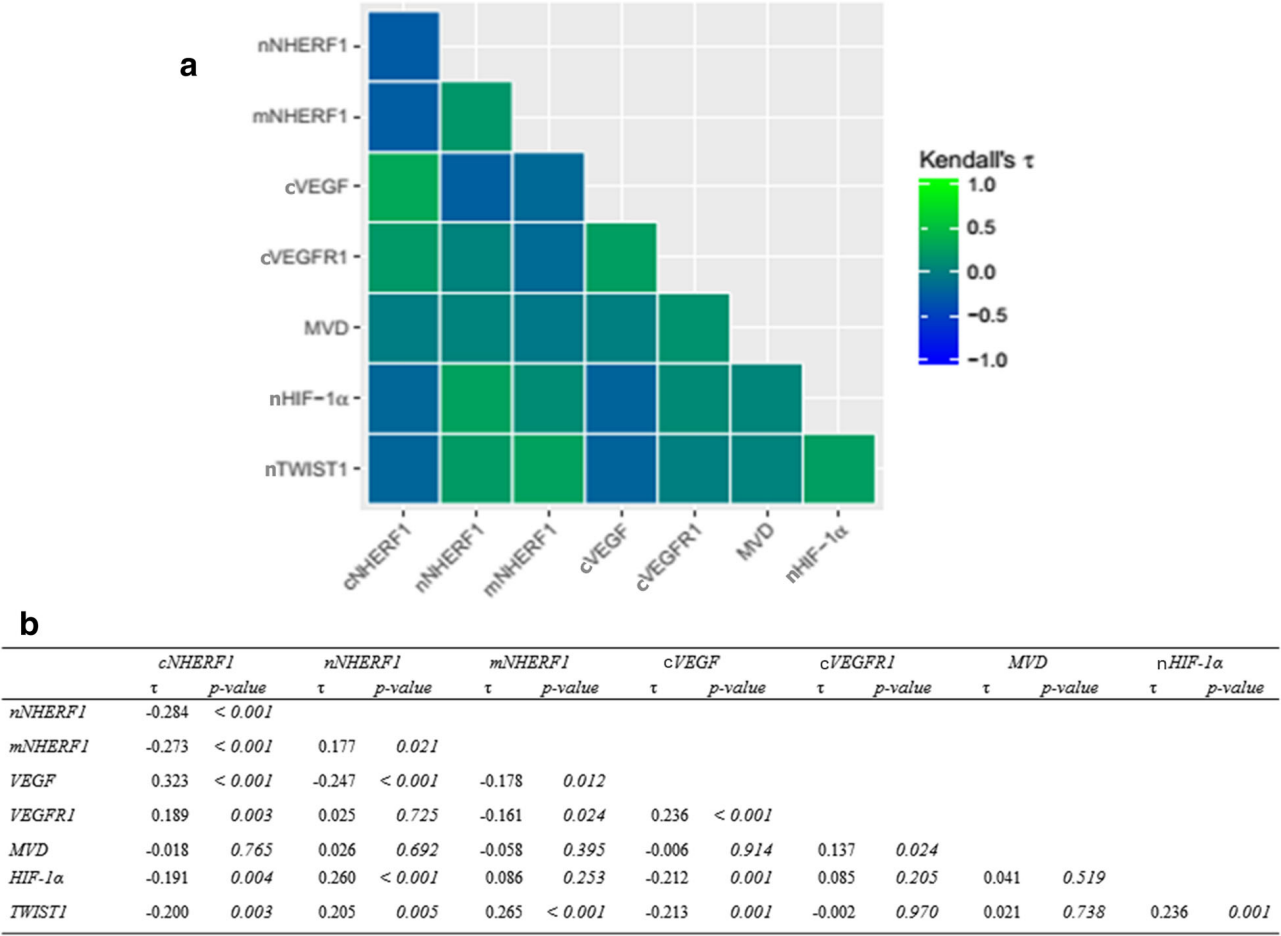
Protein interaction. **a** Heatmap of the protein-protein interaction. **b** The correlation between protein expression of NHERF1 and TME biomarkers was evaluated by Kendall’s τ for rank-based correlations

As regards the dichotomized variables, Chi-squared test for protein associations is summarized in Table 3. For the continuous variables, Kendall’s τ for rank-based correlations between markers and relative heatmap are shown in Fig. 2a and b.

**Table 3:** Relationship between protein expressions in breast cancer patients

	*cNHERF1*				*nNHERF1*			*mNHERF1*			*cVEGF*			*cVEGRF1*			*MVD*			*nHIF-1 α*		
	Negative	Positive		*p-value*	Negative	Positive	*p-value*	Negative	Positive	*p-value*	Negative	Positive	*p-value*	Negative	Positive	*p-value*	Negative	Positive	*p-value*	Negative	Positive	*p-value*
	n (%)	n (%)			n (%)	n (%)		n (%)	n (%)		n (%)	n (%)		n (%)	n (%)		n (%)	n (%)		n (%)	n (%)	
*nNHERF1*																						
Negative	51(42.9)	22(73.3)		***0.003***																`		
Positive	68(57.1)	8(26.7)																				
*mNHERF1*																						
Negative	56(43.4)	17(85.0)		***0.001***	107(82.9)	12(60.0)	***0.017***															
Positive	73(56.6)	3(15.0)			22(17.1)	8(40.0)																
*cVEGF*																						
Negative	39(66.1)	27(34.2)		***< 0.001***	40(67.8)	72(91.1)	***0.001***	45(76.3)	75(94.4)	***0.001***												
Positive	20(33.9)	52(65.8)			19(32.2)	7(8.9)		14(23.7)	4(5.1)													
*cVEGFR1*																						
Negative	40(58.8)	32(40.5)		**0.027**	54(79.4)	63(79.7)	*0.960*	56(82.4)	71(89.9)	*0.185*	37(54.4)	27(35.1)	***0.019***									
Positive	28(41.2)	47(59.5)			14(20.6)	16(20.3)		12(17.6)	8(10.1)		31(45.6)	50(64.9)										
*MVD*																						
Negative	32(47.1)	38(50.0)		*0.724*	56(82.4)	59(77.6)	*0.481*	57(83.8)	68(89.5)	*0.317*	28(41.8)	36(47.4)	*0.503*	41(58.6)	31(38.3)	***0.013***						
Positive	36(52.9)	38(50.0)			12(17.6)	17(22.4)		11(16.2)	8(10.5)		39(58.2)	40(52.6)		29(41.4)	50(61.7)							
*nHIF-1α*																						
Negative	36(40.4)	32(60.4)		***0.021***	78(87.6)	34(64.2)	***0.001***	81(91.0)	42(79.2)	***0.046***	34(37.8)	28(54.9)	***0.049***	49(51.6)	22(40.0)	*0.171*	44(48.9)	23(42.6)	*0.463*			
Positive	53(59.6)	21(39.6)			11(12.4)	19(35.8)		8(9.0)	11(20.8)		56(62.2)	23(45.1)		46(48.4)	33(60.0)		46(51.1)	31(57.4)				
*nTWIST1*																						
Negative	18(29.0)	39(57.4)		***0.001***	56(90.3)	49(72.1)	***0.008***	58(93.5)	56(81.2)	***0.035***	21(32.8)	35(54.7)	***0.013***	33(50.0)	32(45.7)	*0.617*	31(49.2)	31(44.9)	*0.623*	51(78.5)	34(50.7)	***0.001***
Positive	44(71.0)	29(42.6)			6(9.7)	19(27.9)		4(6.5)	13(18.8)		43(67.2)	29(45.3)		33(50.0)	38(54.3)		32(50.8)	38(55.1)		14(21.5)	33(49.3)	

*p-value* of the Chi-squared test for the independence of categorical variables. Bold values indicate significance

Of 183 total tumors, 138 could be assessed for both cVEGF and cNHERF1. In particular, among these 79 tumors over-expressed cNHERF1 and 65.8% had high cVEGF expression as well; additionally, of 59 cases with negative cNHERF1, 66.1% had negative cVEGF expression (*p* < 0.001). Cytoplasmic VEGF negative expression was also related to nuclear (*p* = 0.001) and membranous NHERF1 expression (*p* = 0.001) in 91.1% and 94.4% of cases, respectively. Kendall’s τ for rank-based correlation underlined direct relationship between cVEGF and cNHERF1 (τ = 0.323, *p* < 0.001) and inverse between nNHERF1 (τ = − 0.247, *p* < 0.001) and mNHERF1 (τ = − 0.178, *p* = 0.012). One hundred and forty-seven tumors resulted evaluable for both cVEGFR1 and cNHERF1 expression. In particular, 53.7% the tumors showed cNHERF1 over-expression and 59.5% had also high cVEGFR1 expression (*p* = 0.027). No statistically significant correlation was observed between cVEGFR1 and nuclear and membranous NHERF1 expression. Kendall’s τ test for continuous data showed an inverse association between cVEGFR1 and mNHERF1 (τ = − 0.161, *p* = 0.024), while confirming the direct association between cVEGFR1 and cNHERF1 expression (τ = 0.189, *p* = 0.003). Nuclear HIF-1α and cNHERF1 expression was observed in 142 tumors. Low nHIF-1α expression was related to 60.4% of the cNHERF1 positive tumors (*p* = 0.021) while low nuclear and membranous NHERF1 was related to negative nHIF-1α expression (*p* = 0.001 and *p* = 0.046) for 87.6% and 91% of cases, respectively. Kendall’s τ test confirmed the presence of an inverse correlation between nHIF-1α and cNHERF1 (τ = − 0.191, *p* = 0.004) and a positive correlation between nHIF-1α and nNHERF1 (τ = 0.260, *p* < 0.001). Nuclear TWIST1 and cNHERF1 expression were detected in 130 tumors. Nuclear TWIST1 over-expression linked to a low cNHERF1 detection (*p* = 0.001) appeared in 71% of the cases while low expression of nNHERF1 and mNHERF1 was related to low nTWIST1 expression (*p* = 0.008 and *p* = 0.035) in 90.3% and 93.5% of tumors respectively. The data were also supported by Kendall’s τ test, which emphasized a reverse behavior of nTWIST1 and cNHERF1 (τ = − 0.200, *p* = 0.003) and a positive relation between nTWIST1 and nuclear (τ = 0.205, *p* = 0.005) and mNHERF1 (τ = 0.265, *p* < 0.001). No statistically significant association was found between MVD and NHERF1 for both dichotomized and continuous variables.

**Fig. 3:**
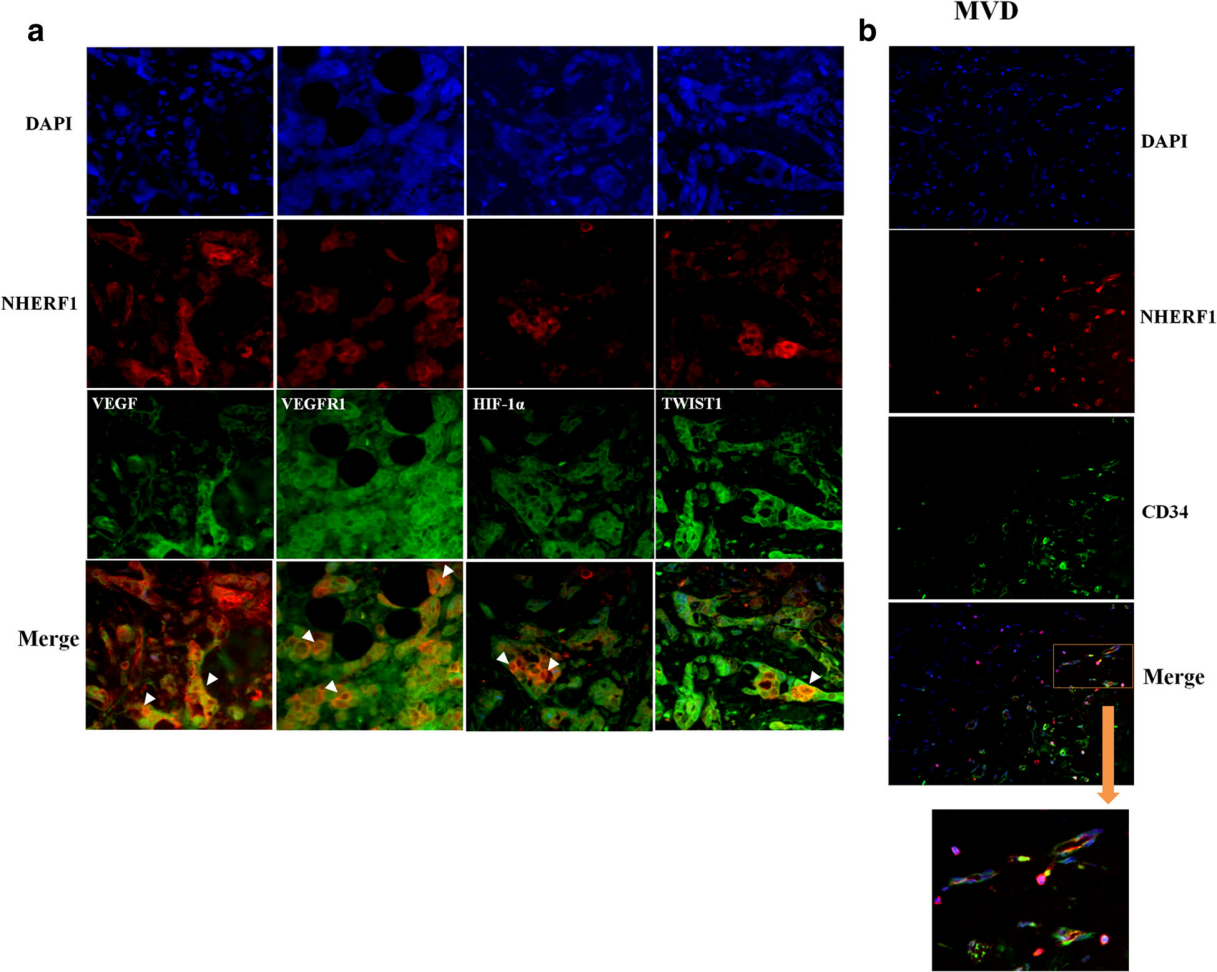
Immunofluorescence analysis of NHERF1 and TME biomarkers expressions in invasive breast cancer. **a** A representative tissue sample stained with NHERF1 and VEGF, VEGFR1, HIF-1α and TWIST1 antibodies and detected with Alexa Fluor 568 (red) and Alexa Fluor 488 (green) secondary antibodies and nucleus in blue (DAPI stained), respectively, prior to fluorescence microscopy analysis (200× magnification). Arrowheads indicate co-localization sites of NHERF1 with the others markers. **b** A representative tissue sample stained with NHERF1 and CD34 antibodies and detected with Alexa Fluor 568 (red) and Alexa Fluor 488 (green) secondary antibodies and nucleus in blue (DAPI stained), respectively, prior to fluorescence microscopy analysis. The square includes microvessel formation with a double staining NHERF1-CD34

The analysis of the localization of VEGF and its receptor VEGFR1, by means of immunofluorescence studies, indicated that they co-localized with NHERF1, when both proteins were over-expressed within cytoplasmic and cytoplasmic and/or membranous compartments respectively, in invasive cellular clusters. Furthermore, our studies indicated that nHIF-1α and nTWIST1 co-localized with NHERF1 too (Fig. 3a). It was interesting to observe that while an immunofluorescence assay showed a high MVD, a neo-vascular formation with a mixed labeling CD34/NHERF1 was also noted (Fig. 3b).

Moreover, of the 183 total tumors, 145 could be assessed for both cVEGF and cVEGFR1, and 64.9% showed a positive relation between cVEGF and cVEGFR1 expression (*p* = 0.019). Of 141 tumor samples valuable for both cVEGF and nHIF-1α, 62.2% of the tumor samples presented cVEGF negative expression and nHIF-1α positive expression (*p* = 0.049). In addition, of the 128 tumors that could be assessed for both cVEGF and nTWIST1, 67.2% of the samples presented cVEGF negative expression and nTWIST1 positive expression (*p* = 0.013). High MVD was associated to cVEGFR1 positive expression (*p* = 0.013) and nHIF-1α/nTWIST1 negative expressions were together related (*p* = 0.001) in 61.7% and 78.5% of the tumors, respectively (Table 3). Furthermore, Kendall’s τ test also revealed a positive correlation between cVEGF and cVEGFR1 expression (τ = 0.236, *p* < 0.001), between cVEGFR1 expression and MVD (τ = 0.137, *p* = 0.024), and nHIF-1α and nTWIST1 expression (τ = 0.236, *p* = 0.001). While, an inverse relation between cVEGF and nHIF-1α (τ = − 0.212, *p* = 0.001), cVEGF and nTWIST1 expression (τ = − 0.213, *p* = 0.001),was observed (Fig. 2b).

### Expression of the proteins and patient outcome

Univariate analyses were carried out for all the clinicopathological characteristics and the expression of cNHERF1, nNHERF1, mNHERF1, cVEGF, cVEGFR1, MVD, nHIF-1α and nTWIST1 proteins, as dichotomized variables. These were correlated to disease-free survival (DFS) and overall-survival (OS) (Table 4). The subgroup of patients with negative expression of nTWIST1 had a shorter 5-year DFS (*p* < 0.001); the same held true for continuous data (*p* = 0.022, data not shown). Moreover, univariate analysis of clinicopathological characteristics in the entire cohort revealed that only MIB1 was significantly associated with worse DFS (*p* = 0.003).

**Table 4:** Univariate analysis with respect to DFS and OS in 118 patients with invasive breast cancer

		DFS				OS		
Characteristics	No. pts.	events	5-year DFS (95% CI)^*a*^	HR (95% CI)^*b*^	*p-value* ^*c*^	events	5-year OS (95% CI)^*a*^	*p-value* ^*c*^
*Overall*	118	37	83 (76, 91)			2	98 (95, 100)	
*Age*								
≤ 49	74	23	84 (75, 94)	1.00	*0.993*	2	97 (92, 100)	*0.275*
> 49	44	14	81 (69, 95)	1.00 (0.51 1.94)		0	100 (100, 100)	
*cNHERF1*								
Negative (< 40%)	48	16	83 (72, 95)	1.00	*0.546*	2	95 (89, 100)	*0.150*
Positive (≥ 40%)	49	15	85 (75, 97)	0.80 (0.40, 1.63)		0	100 (100, 100)	
*nNHERF1*								
Negative (0%)	81	28	83 (74, 92)	1.00	*0.382*	2	97 (93, 100)	*0.547*
Positive (> 0%)	16	3	92 (77, 100)	0.59 (0.18, 1.95)		0	100 (100, 100)	
*mNHERF1*								
Negative (0%)	86	26	85(77, 94)	1.00	*0.133*	2	97 (94, 100)	*0.642*
Positive (> 0%)	13	5	75 (50, 100)	2.06 (0.79, 5.42)		0	100 (100, 100)	
*cVEGF*								
Negative (0–2)	44	12	76 (62, 92)	1.00	*0.632*	1	97 (90, 100)	*0.742*
Positive (3–7)	56	19	88 (79, 97)	0.84 (0.41, 1.73)		1	98 (95, 100)	
*cVEGFR1*								
Negative (0%)	50	16	83 (73, 95)	1.00	*0.427*	1	97 (92, 100)	*0.930*
Positive (> 0%)	56	15	87 (78, 97)	0.75 (0.37, 1.52)		1	98 (94, 100)	
*MVD*								
Negative (< 15microvessels/mm^2^)	42	14	82 (70, 95)	1.00	*0.541*	2	95 (88, 100)	*0.111*
Positive (≥ 15microvessels/mm^2^)	58	16	89 (80, 98)	0.80 (0.39, 1.64)		0	100 (100, 100)	
*nHIF-1α*								
Negative (0%)	62	18	85 (75, 95)	1.00	*0.813*	2	96 (91, 100)	*0.270*
Positive (> 0%)	39	11	91 (81, 700)	0.91 (0.43, 1.94)		0	100 (100, 100)	
*nTWIST1*								
Negative (4%)	46	20	77 (65, 91)	1.00	*< 0.001*	1	97 (92, 100)	*0.304*
Positive (> 4%)	47	8	92 (85, 100)	0.25 (0.10, 0.58)		0	100 (100, 100)	
*Hystological type*								
CDI	105	32	84 (77, 92)	1.00	*0.476*	2	98 (95, 100)	*0.894*
CLI	9	4	59 (92, 100)	1.83 (0.64, 5.18)		0	100 (100, 100)	
Other	4	1	100 (100, 100)	0.72 (0.10, 5.25)		0	100 (100, 100)	
*Hystological grade*								
G1	14	4	91 (75, 100)	1.00	*0.874*	0	100 (100, 100)	*0.240*
G2	53	17	83 (73, 94)	1.30 (0.44, 3.88)		0	100 (100, 100)	
G3	51	16	81 (70, 94)	0.32 (0.44, 3.97)		2	95 (89, 100)	
Tumor s*ize* (cm)								
≤ 2 cm	44	14	93 (85, 100)	1.00	*0.675*	0	100 (100, 100)	*0.242*
> 2 cm	72	21	80 (69, 90)	1.16 (0.59, 2.28)		2	97 (92, 100)	
*Receptor status*								
ER-Negative (≤ 10%)	38	10	80 (68, 96)	1.00	*0.675*	2	94 (85, 100)	*0.033*
ER-Positive (> 10%)	80	27	84 (76, 93)	1.16 (0.56, 2.42)		0	100 (100, 100)	
PgR-Negative (≤ 10%)	47	18	76 (64, 90)	1.00	*0.193*	2	95 (89, 100)	*0.092*
PgR-Positive (> 10%)	71	19	88 (80, 97)	0.65 (0.34, 1.25)		0	100 (100, 100)	
*MIB-1*								
Negative (≤ 20%)	47	7	87 (77, 98)	1.00	*0.003*	1	97 (92, 100)	*0.773*
Positive (> 20%)	71	30	80 (71, 91)	3.20 (1.40, 7.30)		1	98 (96, 100)	
*HER2/neu*								
Negative (0/+ 1)	59	19	88 (79, 98)	1.00	*0.780*	0	100 (100, 100)	*0.098*
Positive (+3)	45	13	75 (61, 90)	1.11 (0.55, 2.24)		2	94 (87, 100)	

^*a*^five-year disease-free survival (DFS) or overall survival (OS) based on Kaplan-Meier method, ^*b*^hazard-ratio (HR) computed using the Cox proportional hazard regression model for the DFS (for OS cannot be computed due to the low number of events), ^*c*^*p-value* of the log-rank test for the equality of probability of an event (relapse for DFS or death for OS)

According to the Cox proportional hazard regression model, multivariate analysis of the entire cohort (Table 5), identified nTWIST1 expression, in both categorical and continuous data (HR = 0,14, 95% confidence interval: CI 0.05–0.43, *p* = 0.001; and HR = 0.92, 95% CI 0.87–0.97, *p* = 0.003, respectively) as significant for DFS. Moreover, for continuous data (HR = 1.09, 95%CI 1.02–1.17, *p* = 0.011), mNHERF1 expression was a significant indicator of DFS (Table 5) as well. HR for OS was not computed due to the low number of events.

**Table 5:** Multivariate analysis with respect to DFS and OS in invasive breast cancer

	categorical		continuous	
Characteristics	HR (95% CI)^*a*^	*p-value* ^*a*^	HR (95% CI)^*a*^	*p-value* ^*a*^
*cNHERF1*	0.63 (0.25, 1.60)	*0.337*	0.99 (0.96, 1.02)	*0.416*
*nNHERF1*	0.60 (0.07, 5.167)	*0.640*	0.89 (0.70, 1.12)	*0.319*
*mNHERF1*	3.71 (0.98, 13.99)	*0.053*	1.09 (1.02, 1.17)	*0.011*
*cVEGF*	0.63 (0.25, 1.61)	*0.333*	0.99 (0.97, 1.01)	*0.249*
*cVEGFR1*	0.64 (0.28, 1.48)	*0.297*	1.00 (0.98, 1.02)	*0.826*
*MVD*	0.54 (0.23, 1.29)	*0.165*	0.97 (0.91, 1.04)	*0.442*
*nHIF-1α*	0.69 (0.24, 2.02)	*0.504*	0.99 (0.95, 1.03)	*0.520*
*nTWIST1*	0.14 (0.05, 0.43)	*0.001*	0.92 (0.87, 0.97)	*0.003*

^*a*^hazard-ratio (HR) and *p-value* for DFS computed using the multivariate Cox proportional hazard regression model with categorical and continuous variables; HR for OS cannot be computed due to the low number of events

**Fig. 4:**
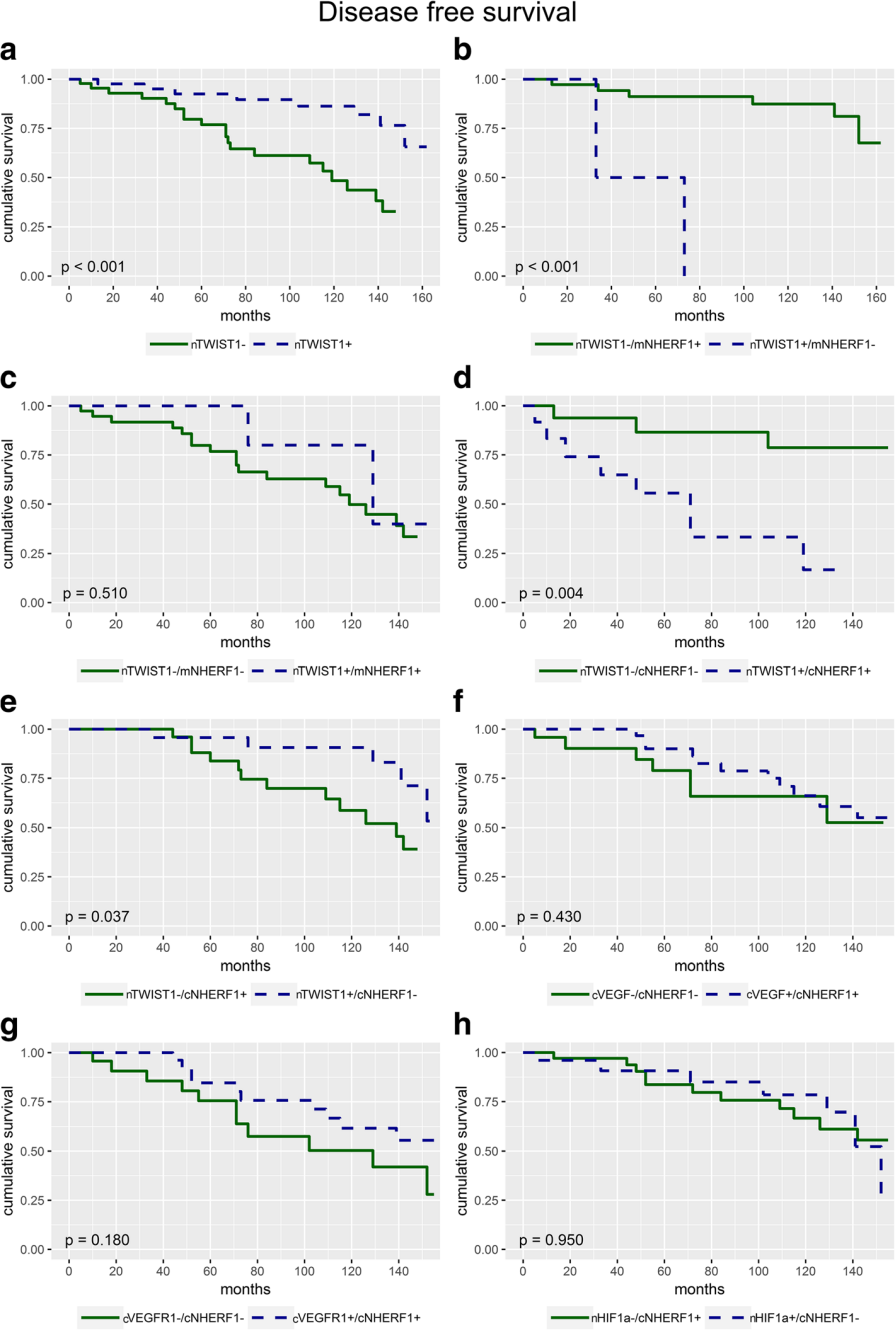
Survival analyses. **a** Disease-free-survival (DFS) curves for patients with nTWIST1+ versus nTWIST1- expression (*p* < 0.001). **b** DFS curves for patients with simultaneously nTWIST1-/mNHERF1+ expression respect to patients with nTWIST1+/mNHERF1- expression (*p* < 0.001). **c** DFS curves for patients with simultaneously nTWIST1-/mNHERF1- expression respect to patients with nTWIST1+/mNHERF1+ expression (*p* = 0.510). **d** DFS curves for patients with simultaneously nTWIST1-/cNHERF1- expression respect to patients with nTWIST1+/cNHERF1+ expression (*p* = 0.004). **e** DFS curves for patients with simultaneously nTWIST1-/cNHERF1+ expression respect to patients with nTWIST1+/cNHERF1- expression (*p* = 0.037). **f** DFS curves for patients with simultaneously cVEGF-/cNHERF1- expression respect to patients with cVEGF+/cNHERF1+ expression (*p* = 0.430). **g** DFS curves for patients with simultaneously cVEGFR1-/cNHERF1- expression respect to patients with cVEGFR1+/cNHERF1+ expression (*p* = 0.180). **h** DFS curves for patients with simultaneously nHIF-1α-/cNHERF1+ expression respect to patients with nHIF-1α+/cNHERF1- expression (*p* = 0.950)

The relationship among the different analyzed biomarkers expression and BCs survival was then investigated. Kaplan-Meier curves revealed that patients with nTWIST1- expression had a worse DFS than of patients with nTWIST1+ expression (5-years 77% vs 92%, *p* < 0.001) (Fig. 4a).

When nTWIST1 and mNHERF1 expressions were considered, Kaplan-Meier curves showed that patients with nTWIST1-/mNHERF1+ immunophenotype had a better DFS respect to patients with nTWIST1+/mNHERF1- (*p* < 0.001) (Fig. 4b). Analyzing patients with nTWIST1-/mNHERF1- vs nTWIST1+/mNHERF1+ expression, no statistically significant results were found (*p* = 0.510), (Fig. 4c).

In addition, the subgroup of patients nTWIST1-/cNHERF1- showed a higher DFS with respect to the subgroup nTWIST1+/cNHERF1+ (*p* = 0.004). When nTWIST1-/cNHERF1+ vs nTWIST1+/cNHERF1- phenotype were compared, the first group showed a worse DFS (*p* = 0.037) (Fig. 4d, e). Kaplan-Meier curves, computed for the other proteins analyzed, revealed that there were no statistically significant results comparing the DFS of the group of patients with cVEGF-/cNHERF1- vs cVEGF+/cNHERF1+ expression (*p* = 0.430) (Fig. 4f), and with cVEGFR1-/cNHERF1- vs cVEGFR1+/cNHERF1+ expression (*p* = 0.180) (Fig. 4g) and nHIF-1α-/cNHERF1+ vs nHIF-1α+/cNHERF1- expression (*p* = 0.950) (Fig. 4h). There were no significant correlations for protein levels and OS.

## Discussion

The study regarding cancer context has become of great significance because of the therapeutic implications linked to it. In recent years, the complexity of TME has been evidenced enough for it to be considered as a real system in an ever-changing state.

In this study, a panel of factors involved in TME changes such as VEGF, VEGFR1, HIF-1a, TWIST1 and MVD, that have been related to BC progression, aggressiveness and invasion was analyzed [6, 12, 28–30]. Further, so as to better characterize this microenvironment, their association with the scaffolding protein NHERF1 and with survival was observed, allowing to identify a possible subgroup of patients that could benefit from a specific therapy.

Mainly, in this BC cohort there was evidence of an increase of cNHERF1 expression with a loss of mNHERF1 detection and a small nNHERF1 presence, in conformity with a more aggressive behavior. Our group had already shown that the switch from apical membranous to cytoplasmic expression was linked to breast carcinogenesis, with a high cNHERF1 expression, present in more aggressive tumors [23]. In addition, the loss of nNHERF1 expression had also been associated with reduced survival, indicating it as an independent prognostic marker in BC [25]. An increase of TME analyzed markers had been reported in these tumors, to confirm their aggressiveness. Moreover, it was shown that cNHERF1 over-expression was related to some aggressive clinical parameters, as demonstrated by association with extensive tumor size and high proliferative activity. Membranous and nuclear NHERF1 expression reaffirmed their protective role, being related to low proliferative activity and smaller tumors. Moreover, our results supported that cVEGF might have a potential additional predictive value, as demonstrated in this population, including younger patients with larger tumors, extensive lymph-node metastases and high grade and proliferative activity. Its receptor cVEGFR1 over-expression was associated to an invasive phenotype as well. VEGF and VEGFR1 have been indicated as strong biomarker indicators to select patients who could benefit from anti-angiogenic therapy [31]. Despite this is an innovative approach, therapeutical failures projected for novel solutions to improve and overcome actual limits [32]. It was e also demonstrated, in this study, that cNHERF1 overexpression was positively related with cVEGF expression. Interestingly, a significant direct association between increased levels of cNHERF1 and cVEGFR1 was also noticed. At the same time, membranous and nuclear NHERF1 reconfirmed their defensive role, being negatively related to cVEGF expression. VEGF is well known as the principal modulator in the process of cancer growth, invasion and metastasis, through new microvessel formation and lymphovascular invasion [33]. Previous studies carried out by our équipe had already reported a NHERF1/VEGFR1 relationship with poor clinical outcome in invasive BCs, indicating a possible interaction between the two proteins [19]. VEGF and VEGFR1 high expression is part of highly hypoxic and aggressive environments, involving high HIF-1α and TWIST1 levels of expression too. Several studies have identified a relation to poorer prognosis/therapy response in the altered expression of HIF-1α and TWIST1 [34–36]. Moreover, the hypoxic environment in BCs is one of the key regulators of the network of the biological elements of matrix, involved in metastasis process and it affects both the early and late stages of metastasis [37]. The dissemination process induced by hypoxia is supplied by paracrine and autocrine stimuli and it is interconnected with EMT program and characterized by high expression of different proteins [38, 39]. The results in this study indicated a direct relation between nHIF-1α and nTWIST1 expression, but a controversial action was present, being both nHIF-1α and nTWIST1 loss interrelated to more aggressive phenotype. Furthermore, an inverse relation was found between cNHERF1 and nHIF-1α and cNHERF1 and nTWIST1 expression, even if a trend consistent with cNHERF1 positive expression was expected, being it correlated to BCs more aggressive phenotype [18, 23, 25]. Additionally, atypically, high nHIF-1α and nTWIST1 expression was present in tumors with low cVEGF expression. Different factors could have contributed to these results, especially knowing that TME is characterized by continuous changing. A possible reason for these discrepancies could be the heterogeneity of BC. Cancer is inherently a heterogeneous disease and so heterogeneity has become its main feature. Clinical and histo-pathological parameters, biomarkers and genetic heterogeneity can be affected by the different characteristics of a single patient (age, menopausal state, general health status etc), but also by epigenetic changes (histone modification, DNA methylation etc) [40, 41] and metabolic reprogramming [42]. Expression of biomarkers can be highly variable within an individual tumor, causing interpretation problems and discordant results.

Regarding nTWIST1 negative expression, it was observed that most of these tumors presented more aggressive clinic-pathological characteristics (high proliferative activity, positive lymph node status, large tumor size, high histological grade and HER2-postive status), despite the fact that there was not a significant statistical evidence for any of these.

Evidence of all this scenario was the presence of new vessels formation, detected by immunohistochemistry and immunofluorescence assay. MVD showed only a direct association with cVEGFR1 over-expression, while no other significant relation has been found. Remarkably, in the tumor area, a neo-vascular formation with a “mosaic” structure was observed with a double staining of CD34 and NHERF1, which could indicate a probable tumor cell recruitment in neo blood vessels formation with endothelial cells. This is a well-known mechanism, which in addition to sprouting angiogenesis, has been recognized to contribute to tumor vascularization. In fact, new blood vessels can be due to vasculogenic mimicry formation, that comprise not only vessels formed by endothelial cells, but also tubules formed by malignant cancer cells [43–45]. NHERF1 involvement in neovascularization phenomena has already been reported [46, 47], but this is the first evidence of a possible direct participation of tumor cells NHERF1 positive to new-vessels formation.

In a subgroup of patients with long-term follow-up, multivariate analysis showed that loss nTWIST1 expression was related to a decrease of DFS. Other authors had reported a worse DFS linked to TWIST1 negative expression, suggesting that its deregulation was involved in poor patient outcome [48, 49]. The Kaplan Meyer curves were used to evaluate the relationship between the biomarkers. Interestingly, when nTWIST1-/mNHERF1+ phenotype were considered, it was discovered that these patients showed a better DFS with respect to nTWIST1+/mNHERF1- phenotype. In addition, data showed a worse outcome in patients nTWIST1-/mNHERF1- vs nTWIST1+/mNHERF1+, despite it being not statistically significant. These findings confirmed the oncosuppressor role of NHERF1 expression, when it was localized at the plasma membrane [50–52], suggesting that mNHERF1 expression might interfere and influence clinical outcome.

Thus, the analyses of nTWIST1+/cNHERF1+ patients selected a phenotype with a worse DFS compared to nTWIST1-/cNHERF1-, as well as the nTWIST1-/cNHERF1+ with respect to nTWIST1+/cNHERF1-, corroborating the pivotal role of cNHERF1 in cancer progression, repeatedly stated by previous researches [18, 23–25].

## Conclusions

In conclusion, the study reported a closer interaction of cNHERF1 with cVEGF, cVEGFR1 and nTWIST1, that denoted an active participation of tumor microenvironment to cancer aggressiveness. From this point of view, it is very interesting to consider the mimicry involving NHERF1^+^ tumor cells in neo-microvessels formation, and this opens a new stimulating scenario for further NHERF1 studies. Moreover, the identification of nTWIST1-/mNHERF1+ phenotype as a subgroup with an increased DFS and nTWIST1+/cNHERF1+, nTWIST1-/cNHERF1+ phenotypes as patient subgroups with poor outcome, reinforced both the tumor suppressor role of mNHERF1 and oncogenic activity of cNHERF1. Furthermore, these date suggest the potential prognostic role of NHERF1. Finally, we believe that our intriguingly results warrant additional studies aimed to evaluate tumor microenvironment regulation in combination with NHERF1 as a possible targeted-oriented therapeutic approach.

## Additional files

Additional file 1: **Table S1.** Dilution, source, staining of antibodies and cut off used. (DOC 44 kb)

Additional file 2: **Table S2.** Expression frequency of biomarkers. (DOC 36 kb)

## Abbreviations

BC: breast cancer
CI: confidence interval
cNHERF1: cytoplasmic NHERF1
cVEGF: cytoplasmic VEGF
cVEGFR1: cytoplasmic VEGFR1
DFS: Disease-free survival
EMT: epithelial to mesenchimal transition
ER: estrogen receptor
HER2: human epidermal growth factor receptor 2
HIF-1α: hypoxia inducible factor-1 alpha
HIS: immunohistochemical score
HR: hazard ratio
IHC: immunohistochemistry
mNHERF1: membranous NHERF1
MVD: microvessel density
NHERF1: Na^+^/H^+^ exchanger regulatory factor 1
nHIF-1α: nuclear HIF-1α
nNHERF1: nuclear NHERF1
nTWIST1: nuclear TWIST1
OS: overall survival
PgR: progesterone receptor
TMA: tissue microarray
TME: tissue microenvironment
VEGF: vascular endothelial growth factor
VEGFR1: vascular endothelial growth factor receptor 1
