# Supplementary material for: NHERF1 and tumor microenvironment: a new scene in invasive breast carcinoma
Source: J Exp Clin Cancer Res. 2018 May 2;37:96. doi: 10.1186/s13046-018-0766-7 (PMC5930748; doi:10.1186/s13046-018-0766-7)
Supplement: Supplementary file 1 — Table S1. Dilution, source, staining of antibodies and cut off used. (DOC 44 kb) [file 13046_2018_766_MOESM1_ESM.doc]

**Additional file 1: Table S1. Dilution, source, staining of antibodies and cut off used**

| **Biomarkers** | **Dilution** | **Source/clone** | **Staining localization** |  | **Cut off (range)** | |
| --- | --- | --- | --- | --- | --- | --- |
| *Progression biomarkers* | |  |  |  |  |  |
| NHERF1 | 1:150 | Affinity Bioreagents, rabbit polyclonal EBP50, PA1-090 | membrane |  | >0%^*^ | (0-40%) |
|  |  |  | cytoplasmic |  | ≥40%^*^ | (0-70%) |
|  |  |  | nuclear |  | >0%^*^ | (0-70%) |
| TWIST1 | 1:50 | Abcam, mouse monoclonal, Twist2C1a | nuclear |  | >4%^*^ | (0-70%) |
|  | |  |  |  |  |  |
|  | |  |  |  |  |  |
| *Microenvironment biomarkers* | |  |  |  |  |  |
| VEGFR1 | 1:100 | Santa Cruz, rabbit polyclonal Flt1, C-17 | cytoplasmic |  | >0%^*^ | (0-87%) |
| VEGF | 1:50 | Santa Cruz, rabbit polyclonal, A-20 | cytoplasmic |  | ≥3^**^ | (0-7) |
| HIF-1α | 1:50 | Santa Cruz, rabbit polyclonal, H-206 | nuclear |  | >0%^*^ | (0-60%) |
| MVD | 1:50 | Novocastra, anti-CD34, mouse monoclonal, QBEnd/10 |  |  | ≥15^***^ | (5-40) |
|  | |  |  |  |  |  |
|  |  |  |  |  |  |  |

^*^median value; ^**^ immunohistochemical score; ^***^microvessels/mm^2^
