# Supplementary material for: NHERF1 and tumor microenvironment: a new scene in invasive breast carcinoma
Source: J Exp Clin Cancer Res. 2018 May 2;37:96. doi: 10.1186/s13046-018-0766-7 (PMC5930748; doi:10.1186/s13046-018-0766-7)
Supplement: Supplementary file 2 — Table S2. Expression frequency of biomarkers. (DOC 36 kb) [file 13046_2018_766_MOESM2_ESM.doc]

**Additional file 2: Table S2. Expression frequency of biomarkers**

| **Biomarkers** | **Tumor samples (N=183)** |
| --- | --- |
|  | **N (%)** |
| **mNHERF1** |  |
| Negative  Positive  not evaluable | 131 (87)  20 (13)  32 |
| **cNHERF1** |  |
| Negative  Positive  not evaluable | 78 (51)  76 (49)  29 |
| **nNHERF1** |  |
| Negative  Positive  not evaluable | 119 (80)  30 (20)  34 |
| **cVEGF** |  |
| Negative  Positive  not evaluable | 69 (46)  81 (54)  33 |
| **cVEGFR1** |  |
| Negative  Positive  not evaluable | 81 (50.3)  80 (49.7)  22 |
| **nHIF1α** |  |
| Negative  Positive  not evaluable | 97 (63.4)  56 (36.6)  30 |
| **nTWIST1** |  |
| Negative  Positive  not evaluable | 68 (49)  72 (51)  43 |
| **MVD** |  |
| Negative  Positive  not evaluable | 72 (46)  85 (54)  26 |
